# Vascularization from Flexible Imaging Color Enhancement (FICE) for polyp localization

**Published:** 2017

**Authors:** VBS Prasath

**Affiliations:** * Computational Imaging and VisAnalysis (CIVA) Lab, Department of Computer Science, University of Missouri-Columbia, USA

**Keywords:** FICE, virtual chromoendoscopy, vascularization, colorectal polyps, localization

## Abstract

**Background.** Virtual chromoendoscopy is an imaging technology that helps in better visualizing the gastrointestinal tract effectively. Recently, the flexible imaging color enhancement (FICE) technique developed by Fijifilm has been considered an alternative to traditional dye spraying. This results in a clearer visualization of the vascular patterns than through the traditional white light endoscopy imaging methods. The performance of vasculature based polyp localization in both traditional and corresponding virtual coloration by FICE was analyzed.

**Conclusion.** Our analysis showed that FICE based images vascularization features provides a better discrimination of polyps than the traditional white light endoscopy images.

## Introduction

Digital chromoendoscopy is an advanced imaging technology that is available for digestive endoscopy and which helps gastroenterologists obtain an unprecedented visualization of the gastrointestinal (GI) tract. 

**Fig. 1:**
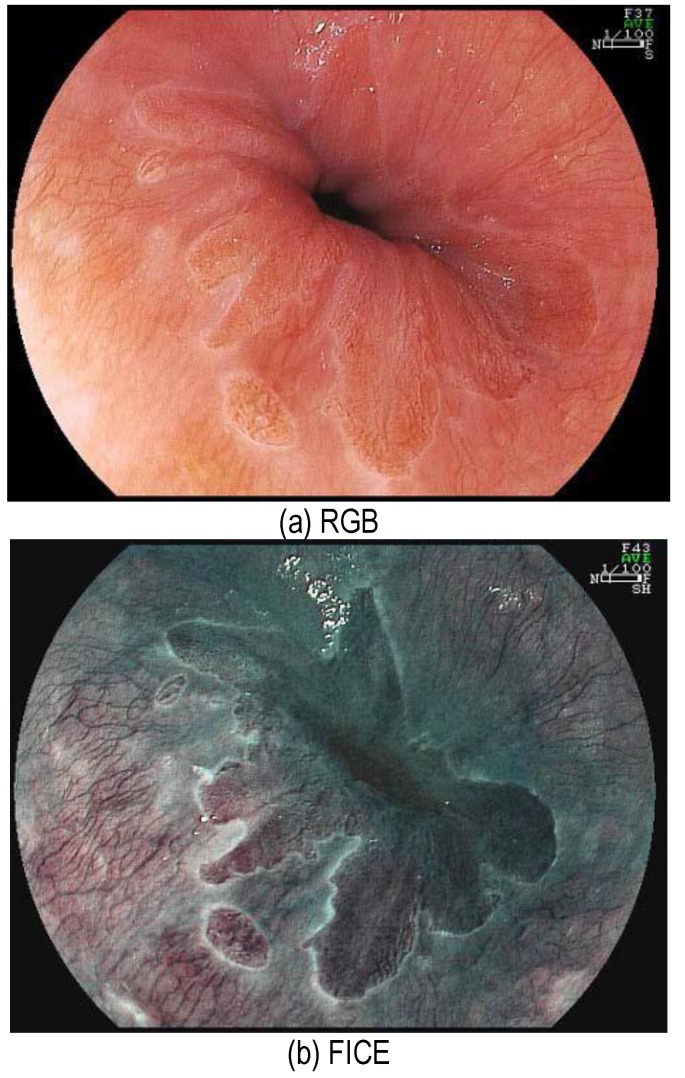
FICE provides improved visualization. (a) Conventional, and (b) FICE. From FICE ATLAS Fujinon

Recently, Fujifilm introduced a new imaging technique called flexible imaging color enhancement (FICE), that provides an alternative color modeling of the endoscopic images via virtual colorization, (see **Fig. 1** for an example), which (a) is the conventional white-light image of the gastroesophageal junction, and (b) is the FICE enhanced image. FICE imaging is implemented based on the spectral estimation technology that takes a white light ordinary endoscopy image process, estimates, and produces an image of a given wavelength of light. FICE facilitates an improved visualization of mucosal linings in the GI tract, and is less invasive than the classical dying utilized in endoscopy [**1**], though there are still some concerns about FICE use in inflammatory bowel disease (see reference [**2**] for a recent review).

Colorectal polyps’ detection is an important task in GI inspection and recently, various image based assistive techniques have been proposed [**3**]. One of the most important features of recognizing polyps and especially of discerning between benign hyperplasias and malignant adenomas is the pit patterns variations [**4**]. 

Vascularization features, computed by using image based vesselness criteria, could be used to assess the polyps and localize them in endoscopy images.

## Aim

This article underlines whether the image based vascularization features in normal white lighting endoscopy imaging versus FICE coloration show improvement in polyps localization.

## Methods

Adenomatous polyps, which have a strong correlation with malignancy and that, are bigger than one centimeter in diameter, are associated with a greater risk of cancer, and, distinguishing them from benign polyps is important. 

Vascularization features can be used to distinguish polyps from normal mucosal tissues. Vascularization can be quantified by utilizing some simple calculations by image calculations [**4**]. These features are calculated by using principal curvature Vk, a multiscale directional vesselness stamping Vd, and Frangi filter [**5**] response Vf. . The final maximum between Vk and (Vd x Vf) has a higher response on the surface of polyps than the surrounding mucosal areas.

## Results and Discussion

**Fig. 2:**
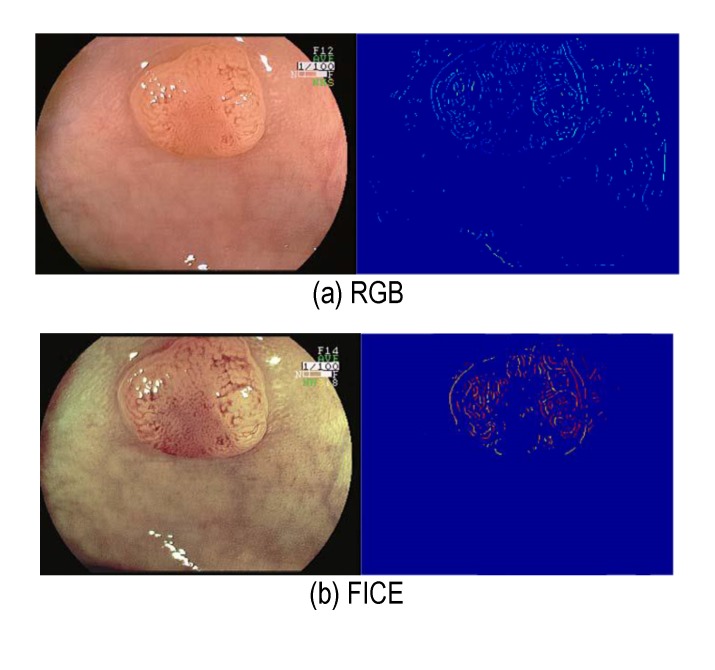
FICE provides improved vascularization on polyp surface. (a) Conventional, and (b) FICE. Top row: Images, bottom row: computed vascularization feature

This maximum vascularization feature between white light endoscopy and FICE enhanced virtual coloration images was tested. **Fig. 2** shows an example of vascularization feature computations for polyp localization in conventional RGB images and FICE versions. As it can be seen from the high values (red regions) in polyp surface, FICE obtains a better localization than the conventional RGB image. This is an adenoma polyp found in the large intestine. The vascular pit pattern is visible to some extent on the conventional low-magnified RGB image, whereas the visualization of the smaller vessels is enhanced on the FICE image. The corresponding vascularization features computations clearly indicate a better localization of this adenomatous polyp. 

It was observed that the vascularization computed from FICE image showed clear high values in the polyp region as compared to the corresponding white light endoscopy image.

**Fig. 3:**
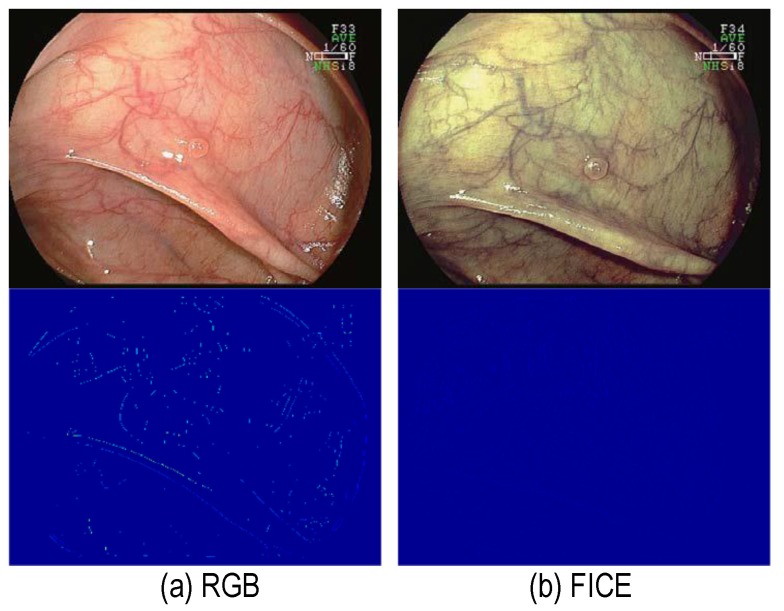
FICE does not alter vascularization features for normal mucosa. (a) Conventional, and (b) FICE. Top row: Images, bottom row: computed vascularization feature

Next, **Fig. 3** evidences a normal mucosa highlighted under conventional RGB imaging and a FICE processed image with the corresponding vascularization feature. As it can be seen, the vascularization values are lower regarding the indication that the localization works only for polyps and not for normal mucosal tissues. This represents a normal mucosa found in the large intestine, with a very small polyp, at the center, that is benign.

**Fig. 4:**
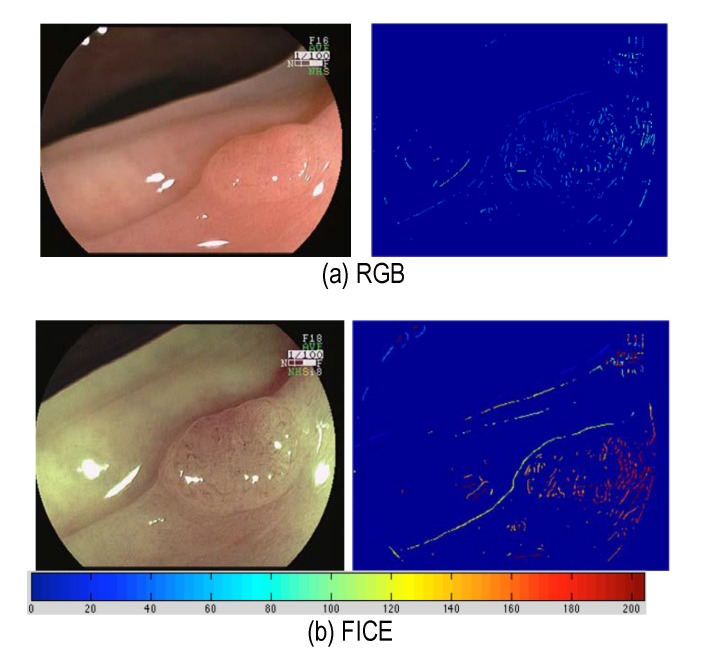
FICE provides an improved vascularization on polyp surface. (a) Conventional, and (b) FICE. Top row: Images, bottom row: computed vascularization feature

**Fig. 5:**
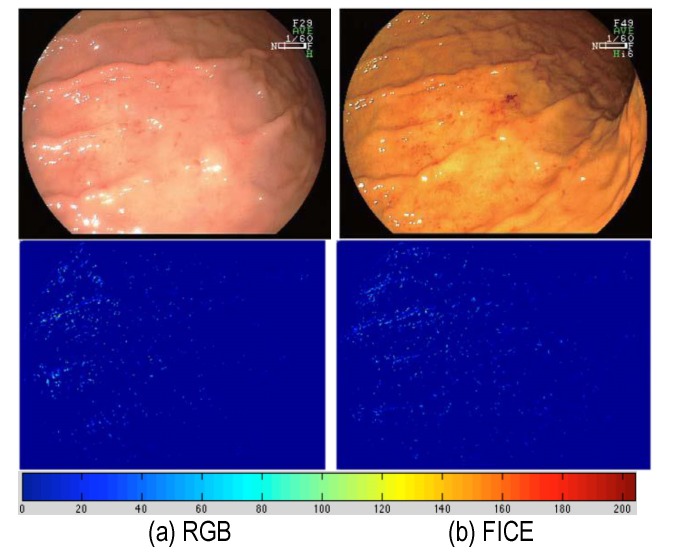
FICE does not alter the vascularization features for the normal mucosa. (a) Conventional, and (b) FICE. Top row: Images, bottom row: computed vascularization feature

In order to further illustrate the advantage of FICE in **Fig. 4** and **Fig. 5**, two examples consisting of an adenomatous polyp and a MALT lymphoma, in the greater curvature of the gastric body, respectively, were shown. As it can be seen, the vascularization features localized well in the adenomatous polyp case, whereas in the lymphoma, the response was lower.

**Acknowledgement**

The images shown in **Fig. 1** are courtesy of Atlas of Spectral Endoscopic Images, Fijifilm, the images in **Fig. 2**-**4** are courtesy of Dr. Kazutomo Togashi, Jichi Medical University, Japan, and the images in **Fig. 5** are courtesy of Dr. Shoji Mitsufuji, Kyoto Prefectural University of Medicine, Japan.

**Conflicts of interest**

The author declares no competing interests.

## References

[1] Omata F, Ohde  S, Deshpande GA, Kobayashi  D, Masuda  K, Fukui  T (2014). Image-enhanced, chromo, and cap-assisted colonoscopy for improving adenoma/neoplasia detection rate: a systematic review and meta-analysis. Scand J Gastroenterol.

[2] Negreanu L, Preda  CM, Ionescu D, Ferechide  D (2015). Progress in digestive endoscopy: Flexible Spectral Imaging. Journal of Medicine and Life.

[3] Prasath VBS (2016). Polyp detection and segmentation from video capsule endoscopy: A review. J Imaging.

[4] Prasath VBS, Kawanaka  H (2015). Vascularization features for polyp localization in capsule endoscopy. IEEE International Conference on Bioinformatics and Biomedicine (BIBM), Proc. IEEE.

[5] Frangi SF, Niessen  WJ, Vincken KL, Viergever  MA (1998). Multiscale vessel enhancement filtering, Medical Image Computing and Computer-Assisted Intervention (MICCAI).

